# Seroprevalence of Human Immunodeficiency Virus, Hepatitis C Virus, and Hepatitis B Virus Among Blood Donors in Sidi Bel Abbes, West Algeria

**DOI:** 10.7759/cureus.47066

**Published:** 2023-10-15

**Authors:** Malika Belkacemi, Mohammed Amine Merbouh

**Affiliations:** 1 Hemobiology and Blood Transfusion, University Hospital Establishment of Oran, Oran, DZA; 2 Medicine, Oran 1 University, Oran, DZA; 3 Epidemiology and Preventive Diseases, Hassani Abdelkader University Hospital, Sidi Bel Abbes, DZA; 4 Medicine, Djilali Liabès University, Sidi Bel Abbes, DZA

**Keywords:** hcv, hbv, hiv, seroptevalence, africain countries, blood donors

## Abstract

Introduction

Blood transfusions can transmit various viruses. Among them, the most common are hepatitis B virus (HBV), hepatitis C virus (HCV), and human immunodeficiency virus (HIV). These viruses can cause fatal and life-threatening diseases. Worldwide, many people are infected with these viruses. Blood safety has made major progress in recent years. Yet, blood-borne viruses continue to be a major concern for patients, physicians, and policymakers. The aim of this study was to assess the prevalence of HIV, HBV, and HCV in blood donors.

Methods

A cross-sectional study was performed to assess HIV, HBV, and HCV seropositivity in blood donors. This research was carried out at the blood bank of Sidi Bel Abbes University Hospital. This was a retrospective study based on records of blood donors. All data of 10,386 donors were analyzed from January 2015 to December 2015. Biological screening was performed by enzyme-linked immunosorbent assay (ELISA) using antibodies and/or antigens. The combined HCV and HIV antigen and antibody ELISA test was utilized. To confirm the results, the blood bank and the virology laboratory used the same technique in duplicate.

Results

The overall seroprevalence of blood-borne viral infections (HIV, HBV, and HCV) in blood donors was 0.8%. The prevalence of HIV was found to be 0.1%, while the prevalence of HBV and HCV was 0.4%. Coinfection was rare with only one case of HBV with HIV. There was a significant difference in seroprevalence rates among blood donors compared to the general population. Significant variations were observed between the prevalence of this study and those conducted in West, East, Central, and South African countries but not with those of neighboring North African countries. The study found no association between seropositivity in blood donors and factors like age, gender, donor status, type of donation, or site. Besides, HIV, HBV, or HCV prevalence was not influenced by ABO and Rhesus blood group.

Conclusion

The study showed that blood donors in Algeria have a lower prevalence of blood-borne viral infections than the general population. The seropositivity rate of viral markers was similar throughout North African countries. This rate remained low compared to other African countries. Residual risk of infection persists. There is a need to increase blood safety for recipients. This report is the first comprehensive overview of blood-borne viruses among Algerian blood donors. There is a need for further nationwide studies to get a whole picture of the situation.

## Introduction

It is now well-recognized that blood transfusions can transmit various viruses. Among them, the most common are hepatitis B virus (HBV), hepatitis C virus (HCV), and human immunodeficiency virus (HIV). These viruses cause fatal, chronic, and life-threatening diseases. HIV, HBV, and HCV infection are major public health issues. Worldwide, approximately 71 million people are infected with HCV, 257 million with HBV, and 36.9 million with HIV, according to recent statistics [1]. People with HBV and/or HCV are at the risk of transition to chronicity with the occurrence of complications such as liver cirrhosis and carcinoma. In 2016, HIV, viral hepatitis, and sexually transmitted infections were reported to be responsible for 1.4 million deaths worldwide [2]. Thus, transfusion-transmitted viruses remain a significant concern for patients, physicians, and policymakers. The vital safety of blood depends on donor selection and screening of the blood for viruses. Over the past few years, blood safety has made great progress by reducing the risk of infection. Besides, we know that blood transfusion around the world meets many regulatory requirements. In Algeria, it is mandatory to screen blood donors for so-called major viruses, which are HIV, HBV, and HCV. These screening tests on blood donors provide essential information on these infections and are part of epidemiological surveillance. The prevalence of these viruses varies by nationality and region [3]. In Algeria, data on transfusion-transmitted viruses are scarce. As such, it is necessary to conduct thorough research in Algeria to determine the prevalence of HIV, HBV, and HCV, among blood donors in the region. Furthermore, it is important to identify factors that are associated with donors, which may contribute to the spread of these infections.

## Materials and methods

Study design, setting, and study population

A cross-sectional study was performed to assess HIV, HBV, and HCV seropositivity in blood donors. This study was carried out at the blood bank of Sidi Bel Abbes University Hospital. It was retrospectively based on the records of successive blood donors from January 2015 to December 2015. Healthy subjects aged between 18 and 60 years and weighing at least 50 kg were eligible for blood donation. The medical and socio-demographic data of the blood donors were recorded. Personal data were not collected in order to preserve the confidentiality of the donor. The Sidi Bel Abbes Hospital University Ethics Committee approved the study (CE/CHUSBA/03/2015).

Laboratory screening test

Screening for HIV, HBS, and HCV markers was done using enzyme-linked immunosorbent assay (ELISA). The search for HIV markers was carried out by the test GenscreenTM ULTRA HIV Ag-Ab (Bio-Rad, France), which detects the HIV p24 antigen and various associated antibodies against the HIV-1 and/or HIV-2 virus. The detection of the viral antigen surface hepatitis B (HBsAg) was performed using the test MonolisaTM Ag HBs ULTRA (Bio-Rad, France). For the serodiagnosis of hepatitis C infection, MonolisaTM HCV Ag-Ab (Bio-Rad, France) was used for the detection of capsid antigens and anti-HCV antibodies. All tests were performed using the BEP 2000 automaton (Siemens). If a serological test yielded negative results, it was deemed that there was no infection. Each positive ELISA test was confirmed by performing the same technique in duplicate in our blood bank and the virology laboratory of the Pasteur Institute in Algiers, using the same or different reagents as those used in the screening.

Statistical analysis

The prevalence of viral markers was determined by the ratio of seropositive people in the total donor population. To express the seroprevalence, a percentage with a 95% confidence interval (CI) was used. Other characteristics of the sample were calculated. The Kolmogorov-Smirnov test of normality was used to analyze the values. Continuous variables are presented as mean and standard deviation (SD) or as median and range (minimum value - maximum value) in the case of not normally distributed data. Paired and unpaired data were compared using the Wilcoxon and Mann-Whitney U test tests. A t-test was used to compare the means between groups. The qualitative variable was introduced in the form of frequencies and percentages. The presence of an association between seropositivity and transfusion data was accessed by Chi-square or Fisher's test and confirmed by binary logistic regression. The odd ratios (ORs) and their 95% CIs were calculated. Statistical analyses were performed in IBM SPSS Statistics software package version 20 (IBM SPSS Armonk NY, USA) and MedCalc Statistical Software version 15.0 (MedCalc Software, Ostend, Belgium). In all statistical tests, the difference was considered significant if the p-value ​​was less than 0.05.

## Results

Through the analysis of the data of 10,386 blood donors, it was found that the sex ratio between males and females was 5.5. The donor data are summarized in Table 1. As expected, there is a disparity between genders in terms of the frequency and the type of blood donation. Besides, we marked a substantial difference between the groups on blood donation sites. We can also observe a difference between men and women in their donor category. Yet, there is no age disparity between women and men. There was no marked difference between the groups about the distribution of the ABO blood group and the Rhesus blood type.

**Table 1:** Characteristics of blood donors at the blood bank of Sidi Bel Abbes University Hospital, West Algeria Chi-square test. Data are expressed as percentage * Mann-Whitney U test. Data are expressed as median value (minimum-maximum) NS: nonsignificant difference

	All	Male	Female	p-value
Number of donors (%)	10,386 (100)	8,783 (84.6)	1,603 (15.4)	
Age (years)	31 (18-65)	31 (18-65)	31 (18-62)	NS*
Donor status (%)				<0.001
First-time donor	1,626 (15.7)	1,268 (14.4)	358 (22.3)	
Regular donor	627 (6.0)	551 (6.3)	76 (4.7)	
Lapsed donor	8,133 (78.3)	6,964 (79.3)	1,169 (72.9)	
Donor category				<0.001
Voluntary donors	3,333 (32.1)	2,695 (30.7)	638 (39.8)	
Replacement donors	7,053 (67.9)	6,088 (69.3)	965 (60.2)	
Blood type donation				<0.001
Whole blood donor	9,766 (94.0)	8,192 (93.3)	1,574 (98.2)	
Platelet pheresis	620 (6.0)	591 (6.7)	29 (4.7)	
Donation site				<0.001
Fixed *site*	8,822 (84.9)	7,531 (85.7)	1,291 (80.5)	
Mobile unit	1,564 (15.1)	1,252 (14.3)	312 (19.5)	
ABO blood group (%)				NS
O	4,703 (45.3)	3,986 (45.4)	717 (44.7)	
A	3,383 (32.6)	2,849 (32.4)	534 (33.3)	
B	1,777 (17.1)	1,506 (17.1)	271 (16.9)	
AB	523 (5.0)	442 (5.0)	81 (5.1)	
Rhesus type (%)				NS
Positive	9,135 (88.0)	7,746 (88.2)	1,389 (86.7)	
Negative	1,251 (12.0)	1,037 (11.8)	214 (13.3)	

In the current study, the overall prevalence was 0.8%. The seroprevalence of HIV was 0.1%, while the individual markers of HBV and HCV had a prevalence of 0.4%. Co-infection was rare, with only one case of HBV with HIV. There was no observed association between HIV and HCV or HBV and HCV. Further statistical tests showed no link between seropositivity for viral markers in a population of blood donors and the following factors such as age, gender, donor status, the type of blood donation, and blood donation site. Besides, HIV, HBV, or HCV prevalence are not influenced by ABO and Rhesus blood group. The results are set out in Table 2.

**Table 2:** Factors related to blood donors associated with HIV, HCV, and HBS seropositivity Chi-square test. Data are expressed as percentage * Mann-Whitney U test. Data are expressed as median value (minimum-maximum) NS: nonsignificant difference, OR: odds ratio, HBV: hepatitis B virus, HCV: hepatitis C virus, HIV: human immunodeficiency virus

			HIV			HCV			HBS	
		Positive	OR (95% CI)	p-value	Positive	OR (95% CI)	p-value	Positive	OR (95% CI)	p-value
Sex (%)		1.643 (0.208-12.979)		NS		1.241 (0.485-3.180)	NS		1.350 (0.531-3.445)	NS
Male	9 (0.1)				34 (0.4)			34 (0.4)		
Female	1 (0.1)				5 (0.3)			5 (0.3)		
Age (years)	34.5 (25-58)			NS	36 (18-55)		NS	30.5 (18-55)		NS*
Donor status (%)				NS			NS			NS
First-time donor	0 (0.0)				8 (0.4)			5 (0.3)		
Regular donor	1 (0.2)				1 (0.2)			5 (0.8)		
Lapsed donor	9 (0.1)				30 (0.5)			32 (0.4)		
Donor category (%)		0.709 (0.200-2.513)		NS		0.843 (0.438-1.624)	NS		1.515 (0.744-3.084)	NS
Voluntary donors	4 (0.1)				14 (0.4)			10 (0.3)		
Replacement donors	6 (0.1)				25 (0.4)			32 (0.5)		
Blood type donation (%)		0.940 (0.936-0.945)		NS		1.314 (0.404-4.279)	NS		1.213 (0.374-3.935)	NS
Whole blood donor	10 (0.1)				36 (0.4)			39 (0.4)		
Plateletpheresis	0 (0.0)				3 (0.5)			3 (0.5)		
Donation site (%)		3.768 (1.062-13.36)		NS		0.644 (0.228-1.814)	NS		0.281(0.068-1.164)	NS
Fixed *site*	6 (0.1)				35 (0.3)			40 (0.5)		
Mobile unit	4 (0.4)				4 (10.3)			2 (0.1)		
ABO blood group (%)				NS			NS			NS
O	4 (0.1)				21 (0.4)			18 (0.4)		
A	4 (0.1)				14 (0.4)			13 (0.4)		
B	1 (0.1)				3 (0.2)			7 (0.4)		
AB	1 (0.2)				1 (0.2)			4 (0.8)		
Rhesus type (%)		1.001(1.000-1.002)		NS		0.931 (0.363-2.385)	NS		1.784 (0.550-5.780)	NS
Positive	10 (0.1)				34 (0.4)			39 (0.4)		
Negative	0 (0.0)				5 (0.4)			3 (0.2)		
Overall	10 (0.1)				39 (0.4)			42 (0.4)		

**Figure 1:**
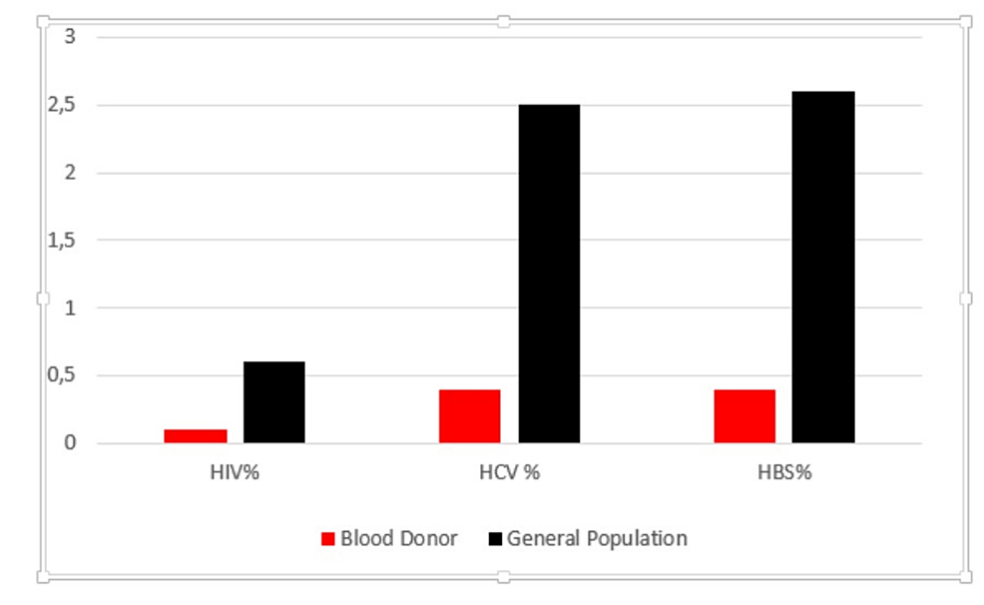
Prevalence of viral markers among blood donors and in the general population

A comparison of the seroprevalence rates in blood donors with the general population is displayed in Figure 1 [4-6]. Looking at this figure, there was a notable disparity between them.

Table 3 provides an overview of the prevalence of viral markers among blood donors in this study and other reports. The results showed that the seroprevalence found in this study was like the rates found in North African nations. On the other hand, there were marked variations between the prevalence found in this study and those recorded in certain countries of west, east, central, and south of Africa.

**Table 3:** The prevalence of viral markers among blood donors in this study and other reports from African countries NS: nonsignificant difference, CI: confidence interval

Country	Region	Number of blood donors	Overall prevalence % (95% CI)	p-value	Reference
Algeria	North Africa	10,386	0.80 (0.63-0.97)		Current study
Morroco	North Africa	25,661	0.60 (0.51-0.69)	NS	[7]
Tunisia	North Africa	19,783	1,80 (0.62-1.98)	NS	[8]
Libya	North Africa	5,802	1.22 (0.94-1.50)	NS	[9]
Egypt	North Africa	6,599	2.20 (1.84-2.56)	NS	[10]
South Africa	Southern Africa	397,640	1.82	NS	[11]
Zimbabwe	Southern Africa	1,586	0.76 (0.34-1.18)	NS	[12]
Namibia	Southern Africa	24,761	0.93 (0.81-1.05)	NS	[13]
Mozambique	Southern Africa	2,783	9.41 (8.33-10.49)	0.0123	[14]
Malawi	Southern Africa	34,437	6.49 (6.23-7.75)	0.0500	[15]
Mali	West Africa	8,207	19.2 (6.84-9.22)	<0.0001	[16]
Negeria	West Africa	2,004	8.03 (6.84-9.22)	0,0319	[17]
Mauritania	West Africa	1,123	13.10 (11.13-15.07)	0,0003	[18]
Burkina Faso	West Africa	4,520	25.86 (13.1-38.62)	<0.0001	[19]
Guinea	West Africa	2,937	21.55 (6.68-36.42)	< 0.0001	[20]
Ghana	West Africa	808	19.70 (16.96-22.44)	< 0.0001	[21]
Djibouti	East Africa	9,006	12.5 (11.82-13.18)	0.0015	[22]
Ethiopia	East Africa	6,827	13.37 (12.56-14.18)	0.0009	[23]
Kenya	East Africa	594	13.5 (10.75-16.25)	0.0027	[24]
Tanzania	East Africa	1,599	14,10 (12.4-15.8)	0.0009	[25]
Congo	Central Africa	599	1.33 (0.41-2.25)	0.0029	[26]
Cameroon	Central Africa	543	18.97 (15.69-22.25)	0.0001	[27]
Gabon	Central Africa	9,992	15.32 (14.62-16.02)	0.0002	[28]
Angola	Central Africa	6,613	16.20 (15.31-17.09)	0.0001	[29]

## Discussion

The purpose of this study was to estimate the seroprevalence of HIV, HCV, and HBV in a university hospital blood bank in Sidi Bel Abbes, a city which is located in the western part of Algeria.

Based on our sample, this study revealed the specificity of characteristics of our blood donor population. Men make up the majority (85%) of the blood donors who come to the blood bank. This finding can be explained by the fact that women, especially those of childbearing age, often suffer from anemia [30]. Therefore, they are ineligible to donate blood. The most common type of blood donation (85%) was whole blood donation. Plateletpheresis makes up 6% of all blood donations. These findings are in line with the data that we have previously published [31]. The majority of blood donors were lapsed donors. Regular voluntary donors represented only a minority. Overall, 16% were first-time blood donors. These findings are consistent with published data [32]. Male donors were more likely to be repeat donors. This result closely fits with what was found previously [33]. Besides, replacement donations accounted for 68% of the total. Yet, a third of donations came from voluntary donors. These findings are in keeping with previous research that has noted a rise in the number of replacement donors [25,34]. Most of the blood donations (84.9%) came from a fixed site and a few from a mobile unit. These observations are consistent with existing data [35]. Blood group O (45.3) was the most common blood group followed by blood groups A (32.6%), B (17.1%), and AB (5%). Belkacemi et al. [36-38] reported a similar ABO blood group and Rhesus-type distribution among blood donors in Algeria.

This study found that the prevalence of viral markers in blood donations is not influenced by several factors related to the donor. These factors include age, gender, donor status, blood donation frequency, and type and site of blood donation, as well as the ABO and the Rhesus blood group. These results are in contrast with some studies, which have noted that the prevalence was higher in older males, replacement donors, first-time blood donors, and blood donors from mobile sites [39-42]. The lack of an association between blood-borne viral infections and the demographic characteristics of the donors may be due to the low prevalence of viral markers in this study. This could be due to individuals’ self-status awareness about infections in our region. The prevalence of viral markers was not linked to ABO and Rhesus blood groups. These results closely fit with what is found within this research [43].

The viral marker rates obtained among blood donors are approximately lower than those observed in the overall population of Algeria. These findings testified to the effective selection of blood donors in our region. Besides, the use of a combined antigen/antibody screening test has been identified as an alternative to molecular biology in developing countries [7]. It is important to note that systematic screening of virus markers using combined reagents has reduced the risk of virus transmission. Yet, it is crucial to recognize that a residual risk of virus transmission still exists. This risk is mainly linked to donations collected during the window serology period, which occurs before the appearance of biological markers of the infection during the early phase [44]. It has been noted that nucleic acid testing identified a higher rate of acute infection compared to conventional serological testing [45]. Thus, to ensure the safety of blood, it is imperative to employ more advanced and efficient methods. It is worth citing that implementing pathogen inactivation for labile blood components can help to eliminate the residual risk of transmitting infection through transfusions [46]. Moreover, these findings ought to serve as a blueprint for enforcing preventive measures to combat the spread of these viral infections within our country.

This study showed that the overall prevalence of blood-borne virus (HIV, HBV, and HCV) infection in blood donors was 0.8%. The rate of viral markers from the North African countries was similar to the present study. However, the rate found in this study was lower than what has been documented in certain other African regions such as the West, East, South, and Central areas. This outcome may be due to the high prevalence of blood-borne viral infections in these areas. The findings of our study concur with previous observations that HIV, HBV, and HCV prevalence varies significantly across geographic regions [47].

The present study has the limitations of a retrospective study. The main weakness stems from a nonrandomized sample selection. Despite this limitation, the strengths of this study include having the first data from a large sample size on the prevalence of blood-borne viral infections among Algerian blood donors.

## Conclusions

The study showed that blood donors in Algeria have a lower prevalence of blood-borne viral infections than the general population. The seropositivity rate of viral markers was similar throughout North African countries. According to the findings of this study, this rate remained low compared to other countries of the rest of the African continent. Yet, there is still a risk of residual infection. New screening tests that use more advanced and effective methods can reduce the risk of blood transfusion transmitting the virus. The study highlights the need for pathogen reduction technology to increase safe blood supply. This research is the first report to provide a comprehensive overview of blood-borne viruses among blood donors in Algeria. Further nationwide studies are needed to get a complete picture of blood-borne viral diseases among blood donors in our country.
